# Joint Use of Skull Base Surgery in a Case of Pediatric Parotid Gland Carcinoma

**DOI:** 10.1155/2014/158451

**Published:** 2014-05-11

**Authors:** Yuri Ueda, Kiyoaki Tsukahara, Kazuhiro Nakamura, Ray Motohashi, Minoru Endo, Hiroki Sato, Yasuaki Katsube, Mamoru Suzuki

**Affiliations:** ^1^Department of Otolaryngology/Head and Neck Surgery, Tokyo Medical University Hachioji Medical Center, 1163 Tatemachi, Hachioji, Tokyo 193-0998, Japan; ^2^Department of Otolaryngology, Tokyo Medical University, 6-7-1 Nishishinjyuku, Shinjyuku, Tokyo 160-0023, Japan

## Abstract

Parotid gland carcinoma is extremely rare in children. We report a case of pediatric parotid gland carcinoma with extensive infiltration into surrounding tissues including the skin and temporomandibular joint capsule at initial examination. Total resection of the parotid gland was conducted together with skull base surgery and mandibular dissection. The patient was a 14-year-old girl. In addition to the skin and temporomandibular joint, infiltration into the anterior wall of the external auditory meatus and masseter muscle was also seen, and T4N0M0 stage IV parotid carcinoma was diagnosed. Skin was resected together with the pinna, and temporal craniotomy and skull base surgery were performed to resect the temporomandibular joint capsule and external auditory meatus en bloc, and mandible dissection was conducted. Facial nerves were resected at the same time. Level I to level IV neck dissection was also conducted. A latissimus dorsi myocutaneous flap was used for reconstruction. The postoperative permanent pathology diagnosis was high-grade mucoepidermoid carcinoma with a low-grade component. Postoperatively, radiotherapy at 50 Gy alone has been conducted, with no recurrence or metastasis observed for over 4 years.

## 1. Introduction

Parotid gland carcinoma is extremely rare in children, with an annual incidence among those less than 19 years old of 0.8 per million [1]. The treatment of parotid gland carcinoma in children is based on that of adults. In most cases, radical resection may be performed through neck surgery alone even in the case of high-grade or advanced parotid carcinoma. We report a case of pediatric parotid gland carcinoma with extensive infiltration into the surrounding area, including the skin and temporomandibular joint capsule on initial examination. Total resection of the parotid gland was conducted in conjunction with skull base surgery and mandibular dissection.

## 2. Case Presentation

**Figure 1:**
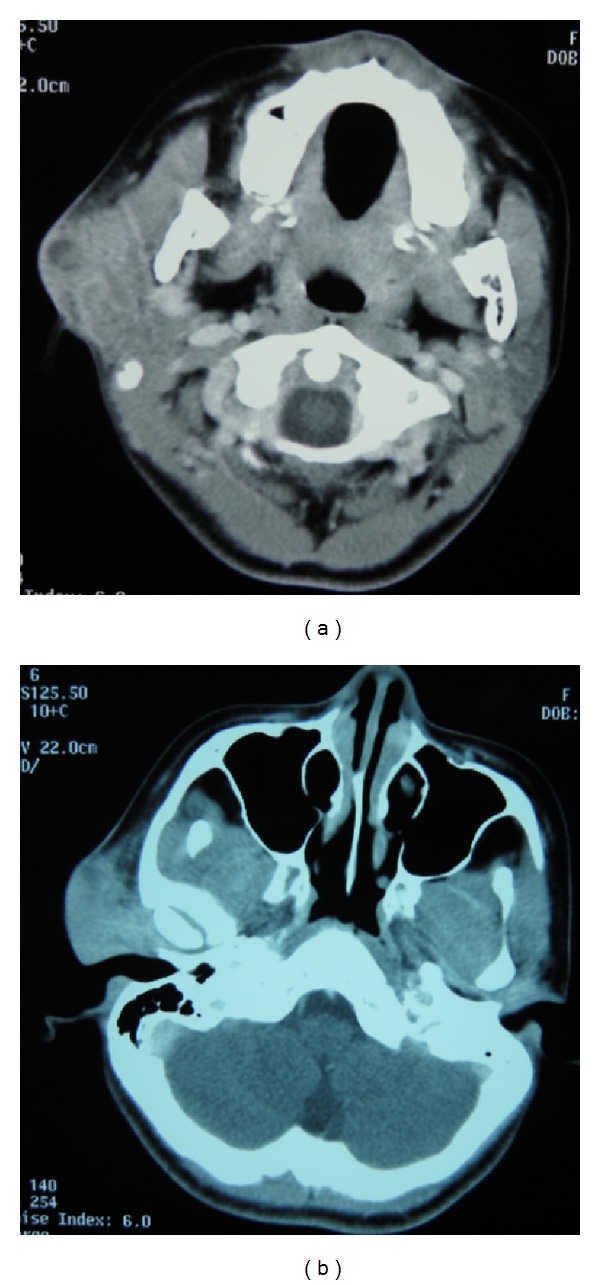
CT findings. (a) A 45 × 40 mm tumor is apparent in the right parotid gland. (b) Infiltration into surrounding tissues is evident, including skin, anterior wall of the external auditory meatus, masseter muscle, and temporomandibular joint.

**Figure 2:**
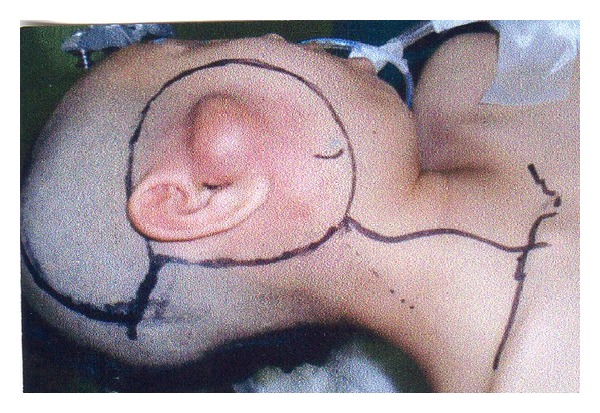
Skin incision. Resection of skin, including pinna.

**Figure 3:**
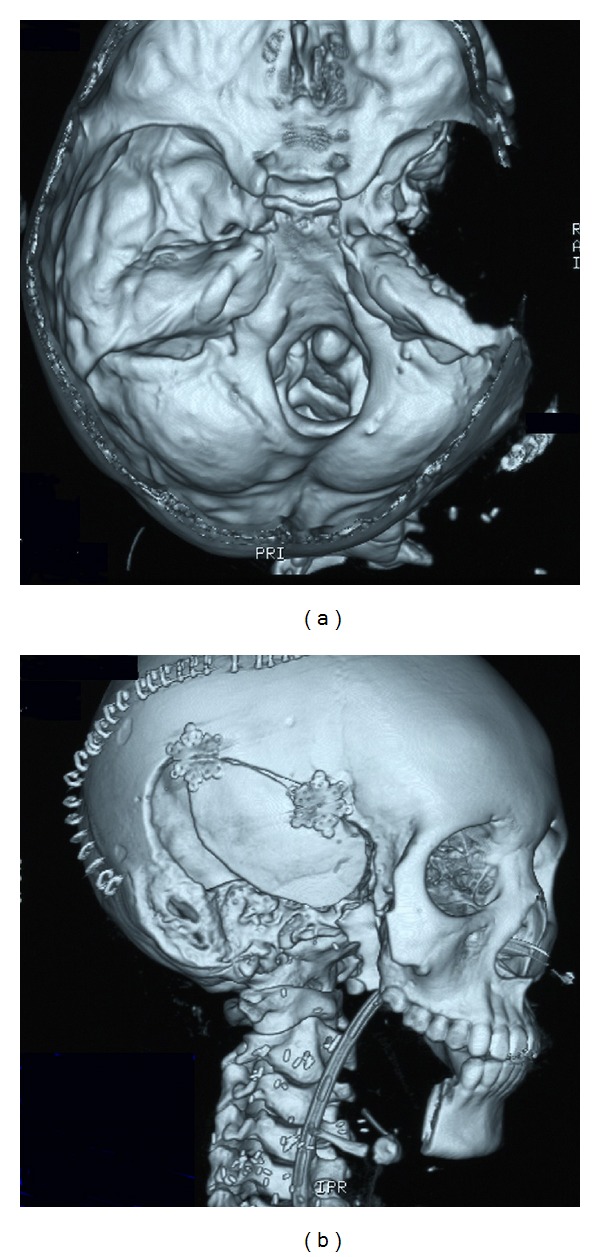
Postoperative 3D CT. The temporomandibular joint capsule, external auditory meatus, and mandibular dissection were removed. The inner ear was retained.

**Figure 4:**
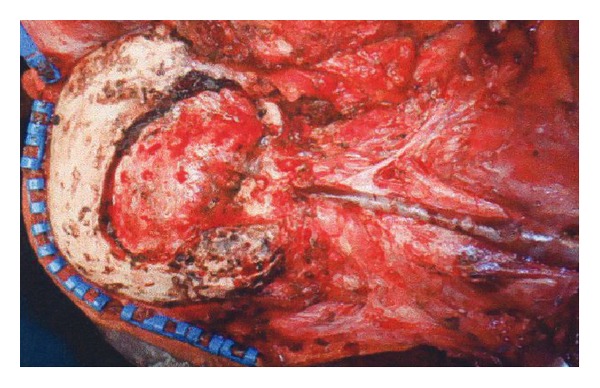
Postresection view. Temporal craniotomy and skull base surgery were performed and mandibular dissection was carried out. Facial nerves were resected at the same time.

**Figure 5:**
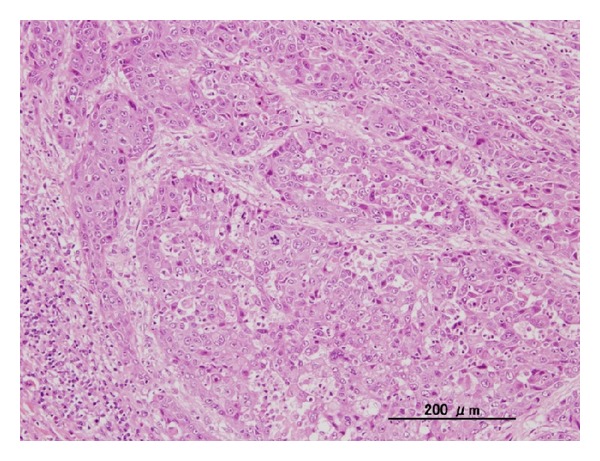
Pathological findings for high-grade malignancy. A large number of strongly heteromorphic, solid foci are apparent.

**Figure 6:**
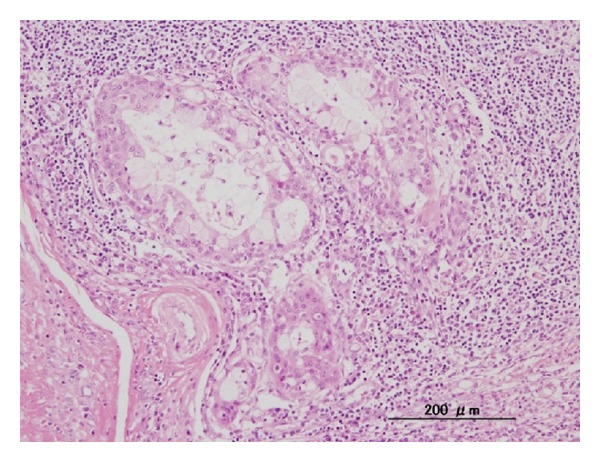
Pathological findings for low-grade malignancy. Ducts formed from goblet cells are clearly distinguishable.

The patient was a 14-year-old girl whose major complaint was a preauricular swelling that had developed 3 years previously. At first, she went to the local doctor and MRI scan was done, but there were no significant findings. Two months before consultation, she had pain around preauricular swelling and visited our hospital. Past history was unremarkable. On initial examination, a massive lesion was observed in the preauricular region with rubor. No facial paralysis was evident. Computed tomography (CT) revealed a 45 × 40 mm tumor in the right parotid gland that had infiltrated surrounding tissues including the skin, anterior wall of the external auditory meatus, masseter muscle, and temporomandibular joint (Figure 1). A CT finding of swellings in multiple lymph nodes in the right neck led us to suspect metastasis into neck lymph nodes, but positron emission tomography- (PET-) CT showed no evidence of metastasis into neck lymph nodes or other parts. Fine needle aspiration revealed large numbers of cell clumps comprising atypical epithelial cells with large, stained ovoid nuclei, and class V high-grade tumor was diagnosed. In view of the above findings, the lesion was considered to be a T4N0 M0 stage IV parotid gland carcinoma. As radical therapy, surgery was performed to resect the skin, including the pinna (Figure 2). Temporal craniotomy and skull base surgery were performed to resect the temporomandibular joint capsule and external auditory meatus en bloc and mandibular dissection was carried out (Figure 3). Dura was retained and facial nerves were resected at the same time. Left neck dissection from level I to level V was also performed (Figure 4). A latissimus dorsi myocutaneous flap was used for reconstruction. Since dynamic reconstruction of facial nerves by means of neuroanastomosis was difficult, static reconstruction was performed. No postoperative complications were encountered. The permanent pathology diagnosis was high-grade mucoepidermoid carcinoma with a low-grade component, in view of the presence of high-grade malignancy with strong heteromorphism and a large number of solid foci (Figure 5) together with low-grade malignancy consisting of clearly visible ducts formed from goblet cells (Figure 6). The resection stump was negative and no metastases to neck lymph nodes were observed. As this was a high-grade mucoepidermoid carcinoma, postoperative radiotherapy at 50 Gy was conducted. In postoperative week 6, the patient was started on a liquid diet, and in week 8 she was able to consume light food. By postoperative week 12, the patient was able to eat normal food again. No recurrence or metastasis has been seen in 4 years of follow-up. Facial deformity, healing loss of the right ear, and occlusion of teeth have remained; however, she was able to eat any foods anywhere with satisfaction. She has enjoyed the university life.

## 3. Discussion

In a study of 113 cases of pediatric major salivary gland malignancy, Shapiro and Bhattacharyya found that mucoepidermoid carcinoma was the most frequent, at 43%, followed by acinic cell carcinoma at 34% and embryonal rhabdomyosarcoma at 6% [2]. Other studies have also noted mucoepidermoid carcinoma as the most frequent type, followed by acinic cell carcinoma [3, 4]. Mucoepidermoid carcinoma accounts for nearly half of parotid gland carcinomas in children and the present case was this type.

Likewise in children, surgery is the main treatment option for parotid gland carcinoma. In the case of low-grade malignancy, preservation of facial nerves and superficial or total parotidectomy are performed and additional treatment is unnecessary. However, if the malignancy is high-grade, more aggressive surgery and auxiliary treatment are required [5]. In the present case, CT revealed that the carcinoma had extensively infiltrated the surrounding area including the skin, anterior wall of the auditory meatus, masseter muscle, and temporomandibular joint. As large numbers of cell clumps comprising atypical epithelial cells with large, stained ovoid nuclei were observed in preoperative cytology, the lesion was considered to be a high-grade carcinoma requiring extensive surgery. In en bloc resection, removal of the temporomandibular joint in one piece was necessary and skull base surgery was performed.

In 26 cases of pediatric skull base surgery, Teo et al. noted that tumors could be completely removed in 92% of cases, with no recurrence in 81% by 2 years postoperatively. However, they observed rates for immediate postoperative complications and permanent complications of 57% and 30.7%, respectively, with spinal fluid leakage and various types of cranial nerve paralysis as the major complications [6]. No postoperative complications were encountered in the present case. Although risks are involved in skull base surgery in children, weighing this against a favorable vital prognosis for its use in extensive surgery to completely remove a tumor, we consider that the surgery should be conducted in the same manner as in adults. Neck dissection has been recommended for high-grade cases, locally advanced cases, and facial nerve paralysis cases even if the patient shows N0 status [7]. As the present case was N0, we followed this recommendation.

For mandibular dissection in adults, reconstruction is conducted using bone transplant. However, for children in the growth phase, stabilization of the bone resection stump by means of a bone transplant can hamper growth of the remaining bone. Therefore, when reconstructing the mandible in growing children, use of transplanted bone is not recommended, other than for defects in the medial region. Also, how the taking of tissue for reconstruction affects donor locations that are in the growth phase remains unclear. In the case of a young female patient like the present, the skin flap option with no bone transplant needs to be selected, in consideration of future childbearing. A latissimus dorsi myocutaneous flap was used for reconstruction in this case. In postoperative week 12, the patient was able to consume normal food. As of the time of writing, 4 years postoperatively at 18 years old, she is able to lead a normal life with no problems, including the ability to exercise freely.

Regarding parotid gland carcinoma in adults, postoperative radiotherapy has been recommended for high-grade cases in which the resection stump is positive or the cancer is advanced [7]. However, as parotid gland carcinoma is rare in children, evidence-based guidelines for postoperative radiotherapy are lacking. Radiotherapy conducted in pediatric patients may cause problems in facial bone development disorders and tooth defects, and the biggest delayed effect is radiation-induced cancer. Use of radiotherapy therefore needs to be limited in such patients. In this regard, the SEER report states that the proportion of patients for whom radiotherapy is conducted is significantly lower for children (27%) than for adults (51%) [1]. In the present case, while no secondary carcinoma or delayed radiotherapy-induced disorders have been encountered, follow-up will need to be continued over the long term.
